# Efficacy of tolvaptan on advanced chronic kidney disease with heart failure: a randomized controlled trial

**DOI:** 10.1007/s10157-022-02224-x

**Published:** 2022-04-26

**Authors:** Shiro Komiya, Mari Katsumata, Moe Ozawa, Tatsuya Haze, Rina Kawano, Yuki Ohki, Shota Suzuki, Yusuke Kobayashi, Akira Fujiwara, Sanae Saka, Kouichi Tamura, Nobuhito Hirawa

**Affiliations:** 1 Department of Medical Science and Cardiorenal Medicine, Yokohama City University Graduate School of Medicine, Yokohama, Japan; 2 Department of Nephrology and Hypertension, Yokohama City University Medical Center, 45-7, Urafune-cho, Minami-ku, Yokohama, 232-0024 Japan; 3 Department of Nephrology, Japanese Red Cross Hadano Hospital, Hadano, Japan; 4 Center for Nobel and Exploratory Clinical Trials (Y-NEXT), Yokohama City University, Yokohama, Japan

**Keywords:** Chronic kidney disease, Heart failure, Tolvaptan, Furosemide, Worsening renal function, Urine osmolality

## Abstract

**Background:**

Tolvaptan (TLV) is reported to improve diuretic effects in patients with chronic kidney disease (CKD) when furosemide (FUR) is not sufficiently effective. However, it is not clear whether TLV addition is effective for advanced CKD patients with heart failure.

**Methods:**

An open-label, parallel-group randomized trial was performed. The subjects were 33 patients with CKD stage G3–G5 who had fluid overload despite taking 20–100 mg/day FUR. They were divided into two groups: a group administered 15 mg/day TLV plus their original FUR dose for 7 days (TLV group), and a group administered 120–200 mg/day FUR (i.e., 100 mg/day over their previous dose) for 7 days (FUR group).

**Results:**

The mean change in urine volume was significantly higher in the TLV group compared to the FUR group (637 ml vs 119 ml; *p* < 0.05). The difference was greater when the urine osmolality before treatment was high. Serum creatinine was increased only in the FUR group. The incidence of worsening renal function (WRF) was significantly lower in the TLV group (18.8% vs 58.8%; *p* < 0.05). Serum sodium decreased significantly in the FUR group, but did not change in the TLV group.

**Conclusions:**

In patients with advanced CKD with fluid overload, the addition of TLV achieved a significantly higher urine volume with less adverse effects on renal function compared with increasing the dose of FUR. The efficacy and safety of TLV were higher in patients who had higher urine osmolality and lower serum sodium before treatment.

**Clinical trial registration:**

UMIN000014763.

## Introduction

Loop diuretics such as furosemide (FUR) have been used to treat fluid overload in patients with heart failure [1]. However, patients with chronic kidney disease (CKD) often do not respond well to loop diuretics [2]. The lower the renal function, the worse is the response to loop diuretics, and increasing the dose of diuretics to improve the response often causes side effects such as deterioration of renal function and electrolyte imbalance [3]. In some cases, dialysis may be required due to inadequate therapeutic effect even after increasing the dose of diuretics.

Tolvaptan (TLV) binds to vasopressin V2 receptors and inhibits water reabsorption in the renal collecting ducts [4]. When used in combination with loop diuretics, TLV has been reported to improve fluid overload [5–7]. Moreover, in recent years, the combination of TLV with loop diuretics has been reported to be more effective than loop diuretics alone in CKD patients [8–12].

However, many of these reports targeted patients with mild-to-moderate CKD. TLV is thought to be less effective as renal function declines [13], and it has not been clarified how effective TLV is in patients with markedly reduced renal function and high diuretic resistance. In our previous retrospective study, we found that TLV was effective in patients with advanced CKD [12], but few studies have prospectively examined the efficacy of TLV in patients with advanced CKD. Therefore, this prospective randomized controlled study was designed to compare the effects of TLV addition versus increased FUR dose in advanced CKD patients. In addition, by examining the factors correlated with those effects, we will examine the indications for future use of tolvaptan.

## Methods

This study was a multicenter, open-label, randomized controlled trial. We enrolled CKD patients in stages G3–G5 with heart failure who were treated at either Yokohama City University Medical Center or Yokohama City University Hospital between July 2015 and July 2020 and who had at least one sign of fluid excess (pleural effusion, ascites, lower leg edema, eyelid edema, pulmonary congestion, or jugular vein distension) despite taking 20–100 mg/day FUR. CKD was diagnosed based on the guidelines of the Japanese Society of Nephrology [14]. Heart failure was diagnosed based on the guidelines of the Japanese Circulation Society [15]. Exclusion criteria included anuria, dialysis, hypernatremia, history of TLV use, hypersensitivity to tolvaptan, and difficulty drinking freely.

The patients were observed for 3 days without changing their FUR dosage. The patients were then randomized into two groups by minimization method using urine volume and serum creatinine levels during the observation period as adjustment factors. The TLV group was treated for 7 days with continued FUR at the baseline dosage plus 15 mg/day TLV. The FUR group was administered 120–200 mg/day FUR (i.e., 100 mg/day over their previous dose). Treatment was inpatient and salt intake was limited to 5 g/day. There was no limit to the amount of water consumed. Urine volume, water intake, and body weight were measured daily, and blood and urine were collected on days 1, 2, 5, and 8 of treatment.

The primary end point of this study was the change in mean urine volume compared to baseline values. Secondary endpoints were change in serum creatinine, incidence of worsening renal function (WRF) (defined as an increase in serum creatinine of 0.3 mg/dl or more from baseline), change in body weight, and change in serum sodium. For urine volume, the mean value during the observation period was defined as the baseline. For other items, the value immediately before the start of treatment was defined as the baseline.

Based on previous reports [12, 16], the change in urine volume (the primary endpoint) was estimated to be + 500 ml/day (SD = 450 ml/day) in the TLV group and + 100 ml/day (SD = 450 ml/day) in the FUR group. We calculated the sample size based on a *t* test assuming equal variances at a two-sided significance level of 5% and a power of 80%, and calculated that 20 patients per group were needed. To account for potential dropouts, the target number of cases was set at 25 cases each, or a total of 50 cases in the two groups.

The full analysis set (FAS) was analyzed. Because the number of missing values was small and missing completely at random, we performed a complete case analysis. Continuous variables were reported as mean ± SD. Continuous variables were compared using the Student’s *t* test for normal distribution and the Wilcoxon rank sum test when the distribution assumption was not met. Linear regression analysis was used to elucidate the relationship between clinical parameters and endpoints. The incidence of WRF was compared by *χ*^2^ test. Patients were divided into two subgroups by median baseline urine osmolality, and changes in urine volume within each group were compared between the two drugs. Multiple regression analysis was used to investigate the effect of drug selection on changes in urine volume. Significance was defined at *p* < 0.05 using two-sided tests. Data analysis was performed using JMP Pro 15.0 (2019; SAS, Cary, NC).

The study protocol was approved by the Ethics Committee of Yokohama City University (D1405025) and was registered at the University Hospital Medical Information Network clinical trial registry (ID: UMIN000014763).

## Results

In all, 35 patients were randomized. One patient met the exclusion criteria and did not receive study treatment. 1 patient was found not to meet the diagnostic criteria for heart failure and was excluded. A total of 33 patients were thus treated and used for analysis. The baseline characteristics of the patients are shown in Table 1. The patients were predominantly male and ranged in age from 44 to 84 years. eGFR was 13.7 and 13.8. The mean dose of FUR prior to randomization was 60 ± 25 mg/day. With the exception of the baseline dose of FUR, the baseline parameters were not significantly different between the two groups.

**Table 1:** Clinical characteristics of patients

	TLV (*n* = 16)	FUR (*n* = 17)	*P* value
Age	66.1 ± 12.2	68.2 ± 10.6	0.589
Sex (male) [*n* (%)]	9 (56.3)	14 (82.3)	0.103
Body weight (kg)	63.6 ± 9.5	69.7 ± 11.5	0.107
Furosemide (mg/day)	50.0 ± 26.3	68.6 ± 21.1	0.030
Duration of furosemide use [*n* (%)]			0.505
≤ 1 week	5 (31.3)	3 (17.6)	
> 1 week, ≤ 3 months	5 (31.3)	6 (35.3)	
> 3 months	6 (37.5)	8 (47.1)	
Average urinary volume for 3 days before administration (ml/day)	1377 ± 540.8	1317 ± 579.1	0.764
Use of ARB, ACEI [*n* (%)]	9 (56.3)	11 (64.7)	0.619
left ventricular ejection fraction	64.5 ± 3.10	60.2 ± 3.01	0.334
Serum creatinine (mg/dl)	4.42 ± 2.33	5.05 ± 2.63	0.468
eGFR (ml/min/1.73 m^2^)	13.7 ± 8.44	13.8 ± 10.2	0.976
CKD stage G4 + G5 [*n* (%)]	16 (100)	16 (94.1)	0.325
Serum sodium (mEq/l)	141 ± 3.01	142 ± 2.45	0.567
Serum osmolality (mOsm/kg)	296 ± 9.48	300 ± 6.57	0.215
Brain natriuretic peptide (pg/ml)	422.8 ± 276.4	520.0 ± 511.9	0.515
Atrial natriuretic peptide (pg/ml)	187.5 ± 115.3	218.0 ± 183.4	0.581
Antidiuretic hormone (pg/ml)	3.2 ± 2.19	2.9 ± 2.43	0.693
Urine osmolality (mOsm/kg)	302 ± 82.7	320 ± 108.4	0.607
Urine specific gravity	1.012 ± 0.004	1.013 ± 0.005	0.645

Data are given as the mean ± SD, or *n* (%)

*eGFR* estimated glomerular filtration rate, *ARB* angiotensin II receptor blocker, *ACEI* angiotensin-converting enzyme inhibitor

Prior to day 7, three patients (19%) in the TLV group and three (18%) in the FUR group discontinued the treatment protocol. Reasons for discontinuation were early completion of treatment due to the resolution of fluid overload (one patient in each group), dry mouth and inadequate efficacy in the TLV group, and blood pressure decrease and patient request in the FUR group.

### Primary end point

**Fig. 1:**
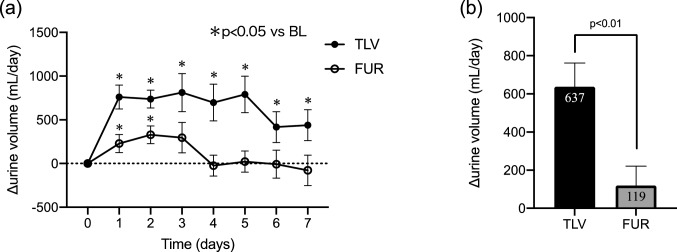
**a** Time course of urine volume during the treatment period compared to the observation period in the TLV group (*n* = 16) and FUR group (*n* = 17). *p < 0.05 vs baseline. **b** Comparison of TLV group and FUR group of average urine volume increase from the observation period

**Fig. 2:**
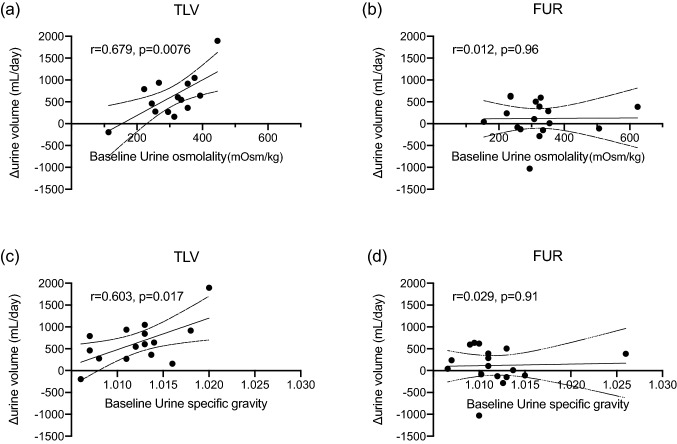
**a** Correlation between urine osmolality at the start of treatment and the change in urine volume from baseline in the TLV group (*n* = 14). **b** Correlation between urine osmolality at the start of treatment and the change in urine volume from baseline in the FUR group (*n* = 17). **c** Correlation between urine specific gravity at the start of treatment and the change in urine volume from baseline in the TLV group (*n* = 15). **d** Correlation between urine specific gravity at the start of treatment and the change in urine volume from baseline in the FUR group (*n* = 17)

**Fig. 3:**
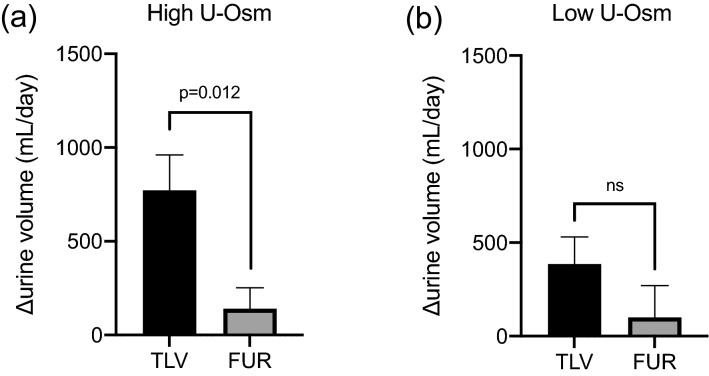
**a** Comparison of changes in urine volume from baseline (ml/day) in the high urine osmolality (> 313.5 mOsm/kg) patients in the TLV and FUR groups. **b** Comparison of changes in urine volume from baseline (ml/day) in the low urine osmolality (< 313.5 mOsm/kg) patients in the TLV and FUR groups

In the TLV group, urine volume per day increased significantly throughout the treatment period. In the FUR group, urine volume increased up to day 2, but did not increase significantly after day 3 (Fig. 1a). The mean change in urine volume over 7 days (or up to the day of early termination) was significantly greater in the TLV group than in the FUR group (*p* = 0.0029; Fig. 1b). Multiple regression analysis showed that the addition of TLV was a factor predictive of increased urine volume (Table 2). In addition, there was a strong positive correlation between baseline urine osmolality and the change in the urine volume from baseline in the TLV group, but not in the FUR group (Fig. 2a, b). Similarly, a strong positive correlation was also observed between baseline urine specific gravity and the change in the urine volume from baseline in the TLV group, but not in the FUR group (Fig. 2c, d). When subjects were divided into two groups by median baseline urine osmolality (313.5 mOsm/kg), the addition of TLV greatly increased urine volume in the group with high urine osmolality, but there was no difference in the group with low urine osmolality (Fig. 3a, b).

**Table 2:** Multiple regression analysis of change in urine volume

Covariate	Standard *β* (95% CI)	*P* value
Tolvaptan	0.449	0.008
Dose of FUR at baseline	− 0.086	0.66
Urine volume at baseline	0.015	0.93
Serum creatine at baseline	− 0.064	0.73

### Secondary end points

**Fig. 4:**
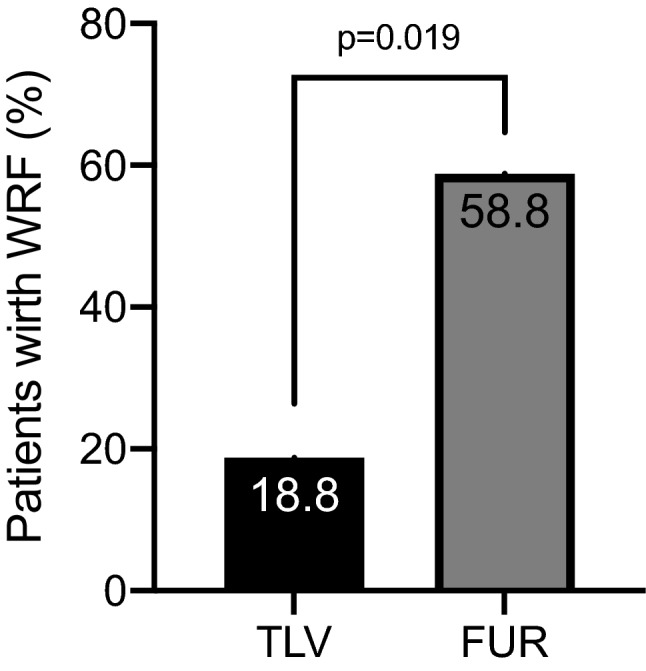
Incidence of worsening renal function in the TLV and FUR groups

Serum creatinine increased from baseline in the FUR group, but not in the TLV group. The incidence of WRF was significantly lower in the TLV group (Fig. 4), with an odds ratio of 0.16 (95% confidence interval 0.03–0.79, *p* < 0.05). The daily weight change (− 0.58 kg/day vs − 0.39 kg/day; *p* = 0.057) and the percentage of weight change for 7 days (− 6.37% vs − 4.17%; *p* = 0.052) showed a trend of decrease in the TLV group compared to the FUR group, but the difference was not significant due to higher water intake in the TLV group (1178 ml/day vs 855 ml/day; *p* = 0.032). Similarly, leg edema tended to resolve more in the TLV group (80.0% vs 46.7% *p* = 0.058). TLV significantly decreased BNP (422.8 pg/ml vs 293.1 pg/ml; *p* = 0.020) and ANP (187.5 pg/ml vs 141.0 pg/ml; *p* = 0.018). FUR also decreased BNP (520.0 pg/ml vs 409.5 pg/ml; *p* = 0.025) and ANP (232.8 pg/ml vs 168.2 pg/ml; *p* = 0.037), with no significant difference between the two groups (BNP: 74.76% vs 79.65%; *p* = 0.86, ANP: 76.56% vs 76.88%; *p* = 0.98).

**Fig. 5:**
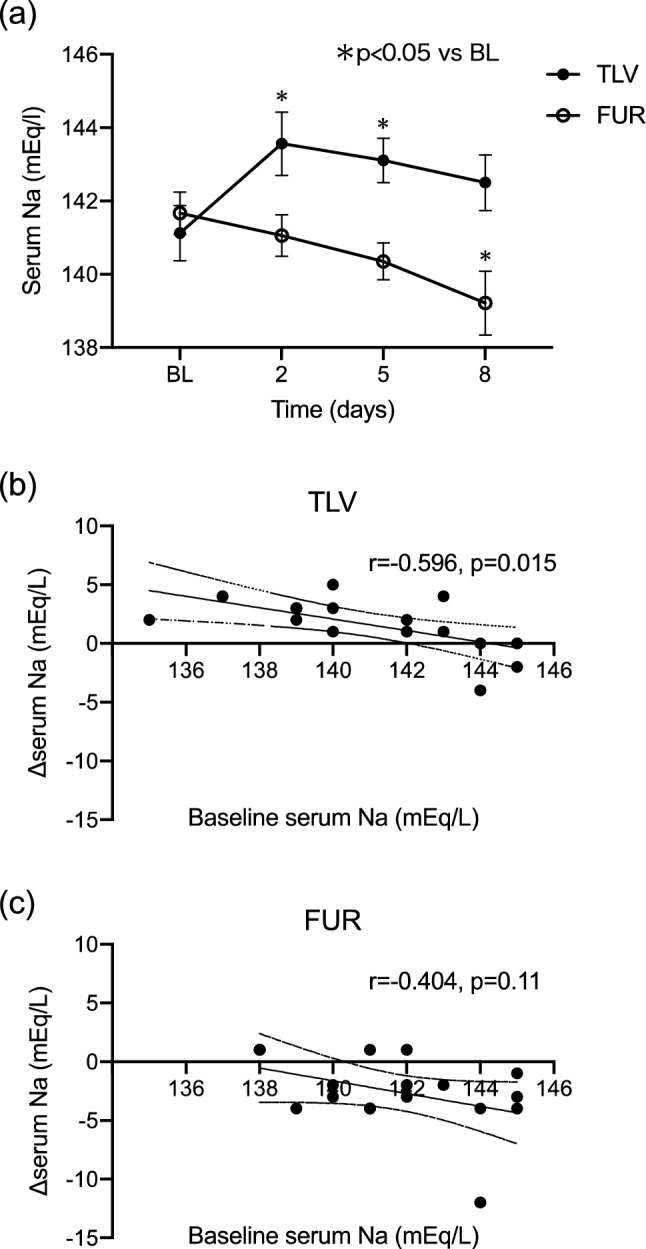
**a** Alterations in serum Na levels before and after treatment in the TLV and FUR groups. **b** Correlation between serum sodium levels at the start of treatment and the change in the serum sodium from baseline in the TLV group (*n* = 16). **c** Correlation between serum sodium levels at the start of treatment and the change in the serum sodium from baseline in the FUR group (*n* = 17)

In the TLV group, serum sodium increased on the day after administration, but then showed a downward trend. In contrast, serum sodium in the FUR group decreased consistently (Fig. 5a). In the TLV group, there was a negative correlation between baseline serum sodium and the change in serum sodium during treatment but not in the FUR group (Fig. 5b).

### Adverse events

One patient with dry mouth and one with hypernatremia were observed in the TLV group, and one patient with hypotension and two with hyponatremia were observed in the FUR group. No serious adverse events were observed.

## Discussion

In patients with advanced CKD who had fluid overload even after treatment with normal doses of FUR, this study showed that the addition of TLV resulted in a greater increase in urine volume and a smaller decrease in renal function than increasing the dose of FUR.

In the conventional comparative studies of TLV and FUR [11, 16, 17], the increase of FUR (often about 20–40 mg/day) in the control group may have been insufficient. Therefore, in this study, the additional dose of FUR in the FUR group was set to 100 mg/day so that the maximum effect of FUR could be expected. Nevertheless, the addition of TLV still achieved a significantly greater increase in urine volume than increasing the dose of FUR.

There was a difference in the baseline dose of FUR between the two groups. However even after adjusting for the baseline dose of FUR in a multivariate analysis, the addition of TLV was an independent factor predictive of increased urine volume. Furthermore, the addition of TLV increased the urine volume irrespective of renal function or responsiveness to FUR.

In our previous retrospective study, we reported that TLV was effective in patients with heart failure who had high urinary osmolality at the start of treatment [12]. Urine osmolality is regulated by ADH. ADH binds to the V2 receptor in the collecting duct of the kidneys and promotes water reabsorption by inducing migration of AQP2 (aquaporin2) to the renal tubule side. ADH is known to be elevated in patients with heart failure, and elevated ADH has been reported to be associated with increased cardiovascular mortality [18]. In patients with heart failure, ADH levels are high despite fluid overload, so it is theoretically possible that TLV, which is a V2 receptor antagonist, would be effective. In particular, patients with high urinary osmolality exhibit relatively insufficient suppression of ADH (excessive ADH), and the effect of TLV is considered to be high in such cases. The present study found that changes in urine volume were strongly correlated with urine osmolality before the start of treatment in the TLV group, but not in the FUR group. Therefore, when we divided patients into two subgroups according to the median urine osmolality, in the group with high urine osmolality, there was a large difference in the change in urine volume between the TLV group and the FUR group (614.1 ml/day, 95% confidence interval 173.4–1054.8), but there was no significant difference in the group with low urine osmolality (285.2 ml/day, 95% confidence interval − 214.5–784.8). From the above, it is considered that TLV addition was more effective than increased FUR dose in increasing the urine volume and expelling excess water from the body, especially in cases in which the urine osmolality at the start of treatment was high.

Measurement of urine osmolality was shown to be useful in the selection of diuretics. However, measuring urine osmolality is often time consuming and may not be measured in the clinic. On the other hand, the urine specific gravity can be measured immediately and easily in the clinic using only a test paper, without the need for special equipment. In this study, we found that the higher the urine specific gravity, the higher the effect of TLV, as in the case of urine osmolality. This would constitute an additional advantage of TLV treatment—namely, its therapeutic effect can be predicted simply by a urine specific gravity test.

It has been reported that renal dysfunction is often associated with the treatment process of fluid excess [19]. WRF may be diagnosed when treatment increases serum creatine levels by 0.3 mg/dl or more, and WRF is known to be a poor prognostic factor [20]. In the treatment of heart failure and renal failure, it is important to stabilize the condition without causing WRF. WRF was thought to be caused by hypoperfusion of the kidneys due to decreased cardiac output and decreased intravascular volume due to diuretic use [21]. However, venous stasis was recently reported to be the factor most associated with WRF [22]. Multiple mechanisms are now thought to contribute to the development of WRF, including initiation of angiotensin II receptor blocker (ARB) / angiotensin-converting enzyme inhibitor (ACEI) [23] and hypotension [24]. Kin et al. reported that in heart failure patients who did not have impaired renal function, treatment with TLV reduced the risk of developing WRF compared with treatment with FUR [25]. Our present study, which included patients with advanced CKD, also found that TLV was less likely to cause WRF than FUR. Whereas FUR mainly reduces extracellular fluid, the combination therapy of TLV and FUR has been reported to reduce intracellular fluid and extracellular fluid as much, resulting in relatively preserved renal blood flow [17]. In addition, several studies [11, 26] have reported that combination therapy improved congestion more, and these may be the factors that prevented the development of WRF. Therefore, TLV was safer, and appeared to improve the prognosis of kidney failure in addition to that of heart failure.

It has been pointed out that patients with heart failure are more likely to develop hyponatremia because they are often treated with diuretics and a low-salt diet [27]. Hyponatremia is considered to be one of the important causes of poor prognosis in patients with heart failure [28], and thus prevention of its onset is important. FUR has been reported to cause hyponatremia in a dose-dependent manner [29], and unfortunately, the serum sodium concentration was significantly decreased by increasing the dose of FUR in this study as well. On the other hand, with the addition of TLV, which is a water diuretic, mild elevation of serum sodium was observed in patients with a tendency toward hyponatremia. Although sufficient caution is required for the development of hypernatremia, no cases with severe hypernatremia were found in this study. Combination treatment with a loop diuretic and TLV can be expected to avoid the hyponatremia observed by treatment with loop diuretics.

## Limitations

This study has several limitations. First, although the study period was extended, the target number of cases was still not reached. There was a tendency for the TLV group to lose more weight and improve leg edema more than the FUR group, but the difference was not significant. Increasing the number of cases may have made a difference. Second, the alterations in BNP and ANP were not significantly different between the two groups. Further studies are needed to determine whether TLV improves heart failure better than FUR. Third, most of the patients were HFpEF patients (88% in the TLV group and 76% in the FUR group). Therefore, we could not determine whether similar results would be obtained in HFrEF patients with advanced CKD. Fourth, since it is also interested in the long-term cardioprotective effect of TLV, but we cannot find the answer because this study examines the short-term effect of TLV on advanced CKD. Long-term studies are required in the future.

## Conclusion

In patients with advanced CKD with fluid overload, addition of TLV achieved a significantly greater increase in urine volume compared to increasing the dose of FUR, and TLV addition did not increase the adverse effects on renal function.

The efficacy and safety of TLV were higher in our patients with higher urine osmolality, and were also higher in our patients with lower serum sodium before the start of treatment.

Patients with advanced CKD usually experience electrolyte imbalance, and their kidneys cannot easily compensate for sudden changes in circulating blood volume. Careful introduction of TLV in patients with CKD with heart failure may improve cardiorenal associations and patient prognosis and quality of life, and thus is an important treatment option.
